# Parental Diabetes Behaviors and Distress Are Related to Glycemic Control in Youth with Type 1 Diabetes: Longitudinal Data from the DINO Study

**DOI:** 10.1155/2017/1462064

**Published:** 2017-12-10

**Authors:** Minke M. A. Eilander, Frank J. Snoek, Joost Rotteveel, Henk-Jan Aanstoot, Willie M. Bakker-van Waarde, Euphemia C. A. M. Houdijk, Roos Nuboer, Per Winterdijk, Maartje de Wit

**Affiliations:** ^1^Department of Medical Psychology, VU University Medical Center, De Boelenlaan 1117, 1081 HV Amsterdam, Netherlands; ^2^Department of Epidemiology and Biostatistics, Amsterdam Public Health Research Institute, Van der Boechorststraat 7, 1081 BT Amsterdam, Netherlands; ^3^Diabeter, Center for Pediatric and Adolescent Diabetes Care and Research, Blaak 6, 3011 TA Rotterdam, Netherlands; ^4^Department of Medical Psychology, Academic Medical Center, Meibergdreef 9, 1100 DD Amsterdam, Netherlands; ^5^Department of Pediatrics, VU University Medical Center, De Boelelaan 1118, 1081 HV Amsterdam, Netherlands; ^6^Department of Pediatrics, University of Groningen, University Medical Center Groningen, Hanzeplein 1, 9713 GZ Groningen, Netherlands; ^7^Department of Pediatrics, Juliana Children's Hospital/Haga Hospital, Els Borst-Eilersplein 275, 2545 AA The Hague, Netherlands; ^8^Department of Pediatrics, Meander Medical Center, Maatweg 3, 3813 TZ Amersfoort, Netherlands

## Abstract

**Objective:**

To evaluate (1) the longitudinal relationship between parental well-being and glycemic control in youth with type 1 diabetes and (2) if youth's problem behavior, diabetes parenting behavior, and parental diabetes-distress influence this relationship.

**Research Design and Methods:**

Parents of youth 8–15 yrs (at baseline) (*N* = 174) participating in the DINO study completed questionnaires at three time waves (1 yr interval). Using generalized estimating equations, the relationship between parental well-being (WHO-5) and youth's HbA1c was examined. Second, relationships between WHO-5, Strength and Difficulties Questionnaire (SDQ), Diabetes Family Behavior Checklist (DFBC), Problem Areas In Diabetes-Parent Revised (PAID-Pr) scores, and HbA1c were analyzed.

**Results:**

Low well-being was reported by 32% of parents. No relationship was found between parents' WHO-5 scores and youth's HbA1c (*β* = −0.052, *p* = 0.650). WHO-5 related to SDQ (*β* = −0.219, *p* < 0.01), DFBC unsupportive scale (*β* = −0.174, *p* < 0.01), and PAID-Pr (*β* = −0.666, *p* < 0.01). Both DFBC scales (supportive *β* = −0.259, *p* = 0.01; unsupportive *β* = 0.383, *p* = 0.017), PAID-Pr (*β* = 0.276, *p* < 0.01), and SDQ (*β* = 0.424, *p* < 0.01) related to HbA1c.

**Conclusions:**

Over time, reduced parental well-being relates to increased problem behavior in youth, unsupportive parenting, and parental distress, which negatively associate with HbA1c. More unsupportive diabetes parenting and distress relate to youth's problem behavior.

## 1. Introduction

In the treatment of youth with type 1 diabetes, the primary caregivers (in most cases parents) play an important role as they perform or monitor the diabetes management on a daily basis. Compared to parents of healthy youth, these parents have more concerns about their child's health. Research showed that parents of youth with type 1 diabetes often worry about severe hypoglycemia (that might result in coma) and the risk of complications later in life [1, 2]. Not surprisingly, increased child-related distress is well described in parent's of youth with type 1 diabetes [3], which influences the parent-child relationship: increased distress is associated with less positive parenting (e.g., positive reinforcement) and more unsupportive parenting behavior (e.g., criticism and nagging) [4]. Within a negative parent-child relationship, youths with type 1 diabetes are more likely to show problem behavior (such as the exhibition of increased symptoms of internalizing and externalizing problems) [5–7] and have less optimal glycemic control [8–10], since parental criticism and nagging discourage youth to manage their diabetes, resulting in increased hemoglobin A1c (HbA1c) levels [11].

Clearly, parenting distress and unsupportive parenting are significant risk factors when it comes to youth's psychological and diabetes outcomes. Supportive parenting could be a significant protective factor for youth's outcomes, as positive communication and supportive parenting empower youth in their diabetes self-care [12, 13].

While it is known that distress is prevalent in the parent's of youth with T1D, little longitudinal research focused on parental well-being. Well-being provides information about generic subjective emotional well-being and quality of life [14]. It is therefore likely to assume that reduced parental well-being is associated with increased parenting distress and negative diabetes-specific parenting and reduced positive parenting behaviors. Also, as previous studies showed that parental distress and negative parenting relate to worsened HbA1c of youth, a negative relation between parental well-being and HbA1c is hypothesized. This has, however, not yet been studied. The current study therefore aims (1) to asses if there is a longitudinal relationship between better parental emotional well-being and more optimal glycemic control in youth and (2) if youth's problem behavior, diabetes parenting behavior, and parental diabetes distress influence this relation. Identification of these mechanisms could help to refine and further develop family interventions to improve outcomes in youth with type 1 diabetes.

## 2. Patients and Methods

### 2.1. Procedure

Data were collected as part of the ongoing Diabetes IN development (DINO) study [15], a longitudinal study on the psychosocial and biological development of youth with type 1 diabetes. Data from three time waves (T0 in 2013, T1 in 2014, and T2 in 2015, each with a 1-year interval) were included in the study reported on in this paper. At T0, T1, and T2, parents completed an online or (in case of nonresponse) a paper survey in which they reported on their own psychosocial functioning and that of their children. Medical data were obtained from hospital charts. Written informed consent was obtained from all parents and from youth ≥ 12 years. The DINO study was approved by the medical ethical committee of VU University Medical Center.

### 2.2. Participants

**Figure 1:**
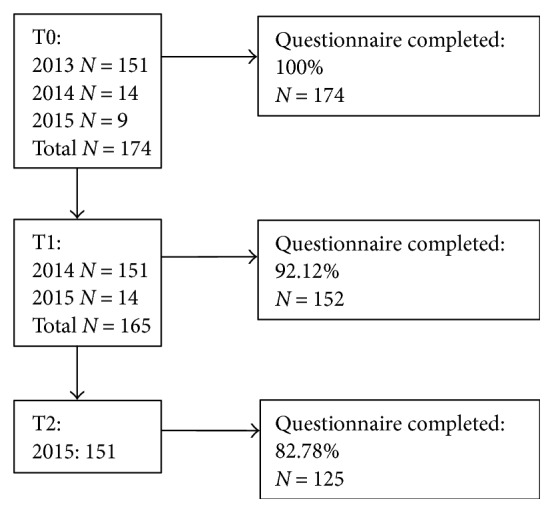
Flowchart participants.

Youth with type 1 diabetes aged 8–15 treated in 5 Dutch diabetes centers and their parents were asked to participate in the DINO study (at the start of the study, 598 youths were invited, in the two years following another 72). Exclusion criteria were as follows: mental retardation, diabetes other than type 1, diagnosis > 6 months, and unfamiliarity with the Dutch language. In total, 25.0% of the invited youths and their parents participated: at the start of the study, 151 were included; one year later, another 14; and in the third year, 9, as shown in Figure 1. At T0, 174 parents (100% of participating parents) completed the questionnaire; at T1, 152 (92.1% of 165 participating); and at T2, 125 (82.8% of 151 participating). The main reasons to reject participation in the ongoing DINO study were inherent to the puberty of youth: they declared to be preoccupied with school and friends, while also managing their diabetes.

### 2.3. Measures

#### 2.3.1. Parental Well-Being

WHO-Five Well-being Index (WHO-5) was used to capture the level of emotional well-being. The scale has five positively worded items, scored on a 6-point Likert scale (e.g., “I have felt cheerful and in good spirits”). Scores are summated with a range from 0 to 25: ≤13 indicates low well-being, and a score of ≤7 likely depression [16, 17]. The WHO-5 is a well-validated questionnaire [14, 17]. Internal consistency for the current sample was 0.92.

#### 2.3.2. Parental Diabetes Distress

The Problem Areas In Diabetes-Parent Revised (PAID-Pr) version was used to assess diabetes-related distress in parents. It contains 18 items, scored on a 5-point Likert scale (e.g., “I feel upset when my child's blood sugars are out of range”). Scores are summated and range from 0 to 72: higher scores indicate more diabetes distress [1]. Internal consistency for the current sample was 0.90.

#### 2.3.3. Diabetes Parenting Behavior

The frequency of supportive and unsupportive diabetes-specific parenting behaviors was assessed using the Diabetes Family Behavior Checklist (DFBC). The DFBC contains 16 items on a 5-point Likert scale, of which nine tap into supportive parenting behaviors (e.g., “Congratulate your child for sticking to their diabetes self-care schedule”) and seven into unsupportive behaviors (e.g., “How often do you argue with your child about his/her diabetes self-care activities”). Scores on the supportive scale range from 9 to 45; a higher total score indicates more parental support. Scores on the unsupportive scale range from 7 to 35; a higher score indicates more criticism and less support [18]. Internal consistency for the supportive subscale for the current sample was 0.64 and for the unsupportive subscale 0.66.

#### 2.3.4. Parental Report of Youth's Problem Behavior

The Strengths and Difficulties Questionnaire (SDQ) was used to measure youth's emotional and behavioral functioning. The SDQ comprises five scales: emotional symptoms, conduct problems, hyperactivity/inattention, peer relationship problems, and prosocial behavior. The parental version of the SDQ was used in this study, in which parents report on their child's behavior. It contains 25 items, scored on a 3-point Likert scale (e.g., “My child often lies or cheats”). Scores are summated and range from 0 to 50: higher scores indicate more problem behavior [19]. The total SDQ score was used in this study. Internal consistency for the current sample was 0.77.

### 2.4. Analyses

Generalized estimating equations (GEE) with exchangeable correlation structure were used to examine the longitudinal relationships between variables. GEE adjust for the correlation between repeated measurements within a person, use all available longitudinal information in analyses, and have the advantage of handling longitudinal data on subjects with varying numbers of unequally spaced observations. The following longitudinal relations were examined: parental well-being on youth's HbA1c; parental well-being on youth's problem behavior; parental well-being on diabetes parenting behavior; parental well-being on parental diabetes distress; diabetes parenting behavior on youth's problem behavior; parental diabetes distress on youth's problem behavior; youth's problem behavior on HbA1c; diabetes parenting behavior on HbA1c; and parental diabetes distress on HbA1c.

We adjusted for baseline report of youth's age, gender, and diabetes duration and parents' gender and education level by including these variables as covariates. *t*-tests were used to examine if parental well-being, diabetes parenting behavior, and parental diabetes distress and youth's glycemic control of dropouts on T1 and T2 differed from parents who did participate on T1 and T2. Data were analyzed using SPSS 22. Parents in a two-parent family decided themselves who completed the questionnaires on each time wave. Therefore, sensitivity analyses using GEE were conducted in which the WHO-5, PAID-Pr, and DFBC scores from deviant parents were excluded from analyses (e.g., when the mother completed the questionnaire at T0 and T2, and at T1, the father was excluded from the sensitivity analyses).

## 3. Results

### 3.1. Participant Characteristics

Baseline characteristics of the 174 parents are presented in Table 1. More mothers than fathers completed the questionnaires (87.3% at T0). The average parental education level [20] was, respectively, high: 64.7%; moderate: 24.1%; and low: 10.4%. The mean age of youth was 11.64 (±2.18) years, and an equal amount of boys and girls participated. Mean HbA1c was 62.15 mmol/mol (±11.35) or 7.84% (±3.19). The mean diabetes duration was 5.10 years (±3.61), 94.2% of youth were born in the Netherlands, and 81.2% lived in a two-parent family. The percentage of parents with WHO-5 scores ≤ 13 indicating low emotional well-being on T0 was 32.0%; on T1, 25.5%; and on T2, 26.7%. Low emotional well-being on more than 1 time point was reported by 22.5% of parents. The percentage of parents with WHO-5 scores ≤ 7 indicating likely depression on T0 was 8.1%; on T1, 10.7%; and on T2, 10.8%. Elevated depressive symptoms on more than 1 time point were reported by 8.1% of parents.

Parental well-being, diabetes parenting behavior, parental diabetes distress, and youth's glycemic control at baseline of dropouts on T1 and T2 did not differ from participating parents (data not shown).

By using GEE, the longitudinal relationships between the variables were analyzed separately, adjusted for parents' education level and gender, youth's age, gender, and diabetes duration, to examine the following: (1) Is there a longitudinal relation between parental well-being and youth's glycemic control? (2) And do youth's problem behavior, diabetes parenting behavior, and parental diabetes distress influence this relation? The results are presented in Table 2.

### 3.2. Parental Well-Being

There was no longitudinal relationship between parental well-being and HbA1c (*β* = −0.052, *p* = 0.650). There was no longitudinal relationship between parental well-being and supportive diabetes parenting behavior (*β* = −0.045, *p* = 0.33) either. However, a longitudinal relationship was found between lower parental well-being on one side and more unsupportive diabetes parenting behavior (*β* = −0.174, *p* < 0.001) and higher parental diabetes distress (*β* = −0.666, *p* < 0.001) on the other. Lower parental well-being was related to increased problem behavior in youth (*β* = −0.219, *p* < 0.001).

### 3.3. Diabetes Parenting Behavior and Parental Diabetes Distress

Unsupportive diabetes parenting behavior and parental diabetes distress were related to worsened glycemic control (*β* = 0.383, *p* = 0.02; *β* = 0.276, *p* = 0.001). Supportive diabetes parenting behavior was related to better glycemic control (*β* = −0.259, *p* = 0.01).

### 3.4. Youth's Problem Behavior

Supportive diabetes parenting behavior did not relate to youth's problem behavior (*β* = −0.013, *p* = 0.82); however, unsupportive diabetes parenting behavior and parental diabetes distress did (*β* = 0.298, *p* < 0.001; *β* = 0.173, *p* < 0.001). Youth's problem behavior was related to poorer glycemic control (*β* = 0.424, *p* < 0.001).

In 13 cases, either T0, T1, or T2 was completed by the other parent (e.g., T0 mother, T1 father, and T2 mother). Sensitivity analyses did not show differences in regression coefficients and significance compared to the primary results based on all data as described above; therefore, these 13 cases were included in the analyses.

## 4. Discussion

The aim of this study was to examine the longitudinal relationship between parental well-being and youth's glycemic control and the influence of youth's problem behavior, diabetes parenting behavior, and parental diabetes distress on this relationship. In our study, about one-third of parents reported low emotional well-being. Reduced parental well-being related to increased problem behavior in youth, more unsupportive diabetes parenting behavior, and parental diabetes distress, which in turn showed a negative effect on glycemic control. Youth's problem behavior was also impacted by unsupportive diabetes parenting and parental diabetes distress. These effects were expected as previous research showed that parenting distress, unsupportive parenting, and youth's well-being impact diabetes outcomes [5, 8, 9]. However, our results did not support the hypothesized direct relation between reduced parental well-being and worsened glycemic control in youth. A relation was found in some [21, 22], but not all cross-sectional studies [4] that examined the association between parental depression and HbA1c. Findings from the current study may differ from previous studies due to differences in measured concepts; we assessed emotional well-being rather than depression. Also, our longitudinal design allowed us to examine the relation over time, providing more robust results. It seems that low parental well-being in general does not relate to HbA1c but it does relate to nonsupportive diabetes parenting behaviors, parental diabetes distress, and youth's problem behaviors, which could affect the quality and the amount of parental involvement in diabetes care, as described by Young et al. [12], which do relate to less optimal HbA1c. This might imply that family interventions aimed to optimize glucose levels are more likely to succeed when they target the reduction of negative diabetes parenting behavior and parental diabetes distress. This in addition to screening for and treating parental low mood and depression, as the effectiveness of such interventions is likely hindered by low parental well-being. The percentage of low well-being in this study is almost ten times higher compared to the normative Dutch population (3.0% and 2.6% of the Dutch population is unsatisfied/unhappy with their life) [23]. Although different measures were used to assess well-being, it confirms previous findings that reduced emotional well-being and depression seem more prevalent in parents of youth with type 1 diabetes [21, 22].

Somewhat surprising, we found no relationship between parental well-being and supportive parenting behavior. Parental well-being seems to relate more to negative than to positive parental functioning. Also, supportive parenting was not associated with less problem behavior in youth. Since supportive parenting was related to better glycemic control, this seems a factor to intervene on in itself.

Currently, several parental interventions that target youth's HbA1c are available, including multisystem therapy, (telehealth) behavior therapy, and parental stress-reduction techniques [24, 25]. The current study is in coherence with previous research when it comes to important aspects of these interventions, namely, the reduction of unsupportive parenting behavior and parental diabetes distress and improving supportive parenting [26, 27]. This is especially true during adolescence when youth become more responsible for their own diabetes tasks [9], a phase in which the communication between parents and youth is challenged. Based on the association between parent's psychosocial aspects and youth's problem behavior as shown in our study, it is likely to assume that due to the reduction of unsupportive parenting and parental diabetes distress, youth's problem behavior improves as well. Our results indicate that intervening on youth's problem behavior might also be beneficial for glycemic outcomes, in line with previous research [28]. Screening for problem behaviors in pediatric diabetes care is recommended by international guidelines [29], although this is challenging in clinical practice [30]. Assessment of parental well-being, diabetes parenting behaviors and distress, and youth's problem behavior seems appropriate within clinical practice, not only to optimize diabetes outcomes but also to improve the quality of life of youth and their parents as well.

Previous literature formed the foundation of the hypothesis that psychological variables influence HbA1c. WHO-5 scores are a reflection of generic and subjective emotional well-being, unrelated to disease [14] and therefore are more likely to be the independent variable. However, it could be argued that there is also a reversed pathway. Law et al., for example, concluded that HbA1c predicts parental distress; nevertheless, cross-sectional data were used in that study [6]. The mechanisms presented in our study can be explained by the cycle of miscarried helping [11]: youth's high or low glucose levels increase parental worries and fear, contributing to negative parent-child communication, characterized by criticism and blame. Consequently, youth feels discouraged and avoids measurement or disclosure of glucose levels, resulting in poor glucose levels. The complexity of the relation between psychosocial and diabetes outcomes is also shown in a longitudinal study revealing that HbA1c was only influenced by parental involvement when youth did not show internalizing behavior symptoms [31]. Clearly, there are other factors that appear to predict and associate with parental functioning, youth's functioning, and diabetes outcomes which were not evaluated in the current study. Diabetes adherence, for example, is found to mediate the relation between critical parenting and HbA1c [8], and less confidence in diabetes self-care and diabetes management mediate the relation between youth's problem behavior and HbA1c [28]. A challenging task for future research is to gain more insight in the complex interactions between these variables. It could be argued that structural equation modeling or mixed models would be appropriate to analyze the longitudinal data presented in this study. However, since the interval between measurements is a year, assumptions about cause (well-being) and effect (HbA1c one year later) would be less reliable. Also, given the sample size, the relatively low frequency of missing data and the varying numbers of unequally spaced observations, GEE were considered most applicable.

### 4.1. Limitations

Although our study contributes to the understanding of the mechanisms between psychosocial factors and diabetes outcomes, there are limitations that we should acknowledge. The main limitation is the low response rate, a quarter of invited families participated. Difficulties in terms of recruiting adolescents with type 1 diabetes are described in other studies as well [10, 32]. This might impact the generalizability of the results. Previous research showed that higher socioeconomic status and being part of an ethnic majority are associated with better diabetes outcomes and emotional well-being [33, 34]. Since diversity in socioeconomic status and ethnicity were lacking in our study, we might have an underestimation of psychosocial problems and poor diabetes outcomes, which highlights the importance of screening for these problems in clinical care. It also stresses the relevance of future studies in more diverse populations. Even though youth's behavior is frequently assessed by parental proxy reports, a recent study showed that parents' self-reported well-being associates with their assumptions of their children's well-being [35]. Based on this parental proxy bias, it seems worthwhile to examine if the relation between parental well-being and problem behavior diminishes when youth's reporting of their own functioning is used. The internal consistency (Cronbach's *α*) of the DFBC proved to be questionable in our study (supportive scale: *α* = 0.64; unsupportive scale: 0.66), which is, however, in coherence with other research (0.73 and 0.43, resp.) [18].

In summary, this longitudinal study showed that reduced parental well-being is associated with unsupportive diabetes parenting behavior, parental diabetes distress, and youth's problem behavior, which in turn relate to less optimal glycemic control. These findings highlight the importance of interventions that not only target youth's glycemic control but also target parents' psychosocial functioning.

## Figures and Tables

**Table 1:** Baseline characteristics.

Boys *N* (%)	87 (50.0)
Youth's age (yrs)	11.64 (2.18)
HbA1c % (mmol/mol)	7.84 (±3.19) (62.15 (±11.35))
Age diabetes onset	6.56 (3.72)
Diabetes duration (yrs)	5.0983 (3.61)
Pump/injections *N* (%)	134/39 (77.5/22.4)
Completion by mothers *N* (%)	151 (87.3)
Completion online/paper *N* (%)	157/16 (90.8/9.2)
Parental education *N* (%)^∗^	
(i) Low	18 (10.4)
(ii) Moderate	42 (24.1)
(iii) High	112 (64.7)
(iv) NA	1 (0.6)
Ethnic identification other than Dutch^∗∗^* N* (%)	10 (5.8)
Adolescents born in the Netherlands *N* (%)	169 (94.2)
Traditional family composition *N* (%)	138 (81.2)
WHO-5 (0–25)	15.49 (5.14)
DFBC supportive (9–45)	30.15 (5.37)
DFBC unsupportive (7–35)	14.45 (4.44)
PAID-Pr (0–72)	13.54 (9.08)
SDQ (0–40)	8.20 (6.03)

Data are means ± SD, unless otherwise indicated; HbA1c: hemoglobin A1c; WHO-5: WHO-Five Well-Being Index; DFBC: Diabetes Family Behavior Checklist: PAID-Pr: Problem Areas In Diabetes-Parent Revised version; SDQ: Strengths and Difficulties Questionnaire; ^∗^low: primary school; LBO, Mavo, VMBO, MBO-1, and avo-onderbouw; moderate: Havo, HBS, VWO, and MBO; high: HBO and university [20]. ^∗∗^Parents were asked with which ethnicity they identified themselves.

**Table 2:** Relations between parental well-being, HbA1c, youth's problem behavior, diabetes parenting behavior, and parental diabetes stress.

	*β*	95% LCI	95% UCI	*p* value
WHO-5—HbA1c	−0.052	−0.275	0.172	0.650
WHO-5—DFBC supportive	−0.045	−0.134	0.044	0.325
WHO-5—DFBC unsupportive	−0.174	−0.268	−0.081	0.000^∗∗^
WHO-5—PAID-Pr	−0.666	−0.858	−0.474	0.000^∗∗^
WHO-5—SDQ	−0.219	−0.312	−0.125	0.000^∗∗^
DFBC unsupportive—HbA1c	0.383	0.069	0.697	0.017^∗^
PAID-Pr—HbA1c	0.276	0.117	0.436	0.001^∗∗^
DFBC supportive—HbA1c	−0.259	−0.455	−0.063	0.010^∗^
DFBC supportive—SDQ	−0.013	−0.123	0.097	0.821
DFBC unsupportive—SDQ	0.298	0.165	0.431	0.000^∗∗^
PAID-Pr—SDQ	0.173	0.104	0.242	0.000^∗∗^
SDQ—HbA1c	0.424	0.191	0.658	0.000^∗∗^

HbA1c: hemoglobin A1c; WHO-5: WHO-Five Well-Being Index; DFBC: Diabetes Family Behavior Checklist; PAID-Pr: Problem Areas In Diabetes-Parent Revised version; SDQ: Strengths and Difficulties Questionnaire; ^∗^Statistically significant at the *p* < 0 .05 level; ^∗∗^statistically significant at the *p* ≤ 0.001 level.
